# Ponatinib confers adult human cardiomyocyte toxicity via inhibition of AKT signaling

**DOI:** 10.14814/phy2.70877

**Published:** 2026-04-17

**Authors:** Linna Guo, Rongjia Rao, Hong Liu, Bingying Zhou

**Affiliations:** ^1^ State Key Laboratory of Cardiovascular Disease, Fuwai Hospital, National Center for Cardiovascular Diseases Chinese Academy of Medical Sciences and Peking Union Medical College Beijing China; ^2^ Shenzhen Key Laboratory of Cardiovascular Disease Fuwai Hospital, Chinese Academy of Medical Sciences Shenzhen China

**Keywords:** cardiotoxicity, human primary cardiomyocytes, PI3Kα‐AKT pathway, ponatinib

## Abstract

As a widely used anticancer drug for the treatment of chronic myeloid leukemia, ponatinib is known to cause severe cardiovascular toxicities. Cardiomyocytes are the major functional units in the myocardium, and impairment of their viability and function plays a crucial role in cardiotoxic responses. Previous studies have indicated direct toxicity of ponatinib on cardiomyocytes, but the effect of ponatinib on adult human primary cardiomyocytes (hPCMs) remains unknown, largely due to sample scarcity and the technical challenges associated with the adult human cardiomyocyte model. Based upon our previous work establishing hPCMs as a pharmacologically competent model, we tested the direct toxicity of ponatinib on these cells. We reveal suppression of AKT, but not ERK, phosphorylation upon ponatinib treatment. Treatment of hPCMs with AKT inhibitor MK‐2206 phenocopied ponatinib, while restoration of AKT signaling with UCL‐TRO‐1938 or insulin partially rescued hPCMs from cell death, suggesting a potential protective effect.

## 1 INTRODUCTION

Drug‐induced cardiotoxicity poses a great challenge to both drug development and clinical application. In particular, cardiotoxicity triggered by anticancer drugs has garnered widespread attention, which has led to the emergence of cardio‐oncology (Gao et al., 2024). While effective at improving the survival rates of cancer patients, these same drugs can also cause adverse effects such as heart failure (HF), myocarditis, vascular toxicity, hypertension, and arrhythmias (Lyon et al., 2022).

In addition to the commonly encountered dose‐dependent cardiotoxicity induced by chemotherapies (e.g., anthracyclines) and radiotherapies (a.k.a. type I toxicity), targeted therapies can also cause toxicity that is considered reversible (type II toxicity). For example, small‐molecule tyrosine kinase inhibitors (TKIs) targeting breakpoint cluster region gene‐Abelson murine leukemia viral oncogene homolog 1 (BCR‐ABL), such as dasatinib, nilotinib, and ponatinib, are known to cause cardiotoxicity while treating chronic myeloid leukemia (CML) (Lyon et al., 2022). Among these, ponatinib exhibits broad‐spectrum TKI activity and therefore likely causes various off‐target activities. In a phase II study of ponatinib involving 449 patients, the cumulative incidence of arterial occlusive events (AOE), including cardiovascular, cerebrovascular, and peripheral vascular events, was 26% (Cortes et al., 2018). Ponatinib induced HF or left ventricular dysfunction at an incidence ranging from 3% to 15% (Chang, Moudgil, et al., 2017). It also led to myocardial infarction or ischemia‐related events, occurring at a rate of up to 12% (Chang, Moudgil, et al., 2017). Furthermore, hypertension was its most common cardiotoxic manifestation, with a reported incidence as high as 68% (Chang, Okwuosa, et al., 2017). Ponatinib can also cause various diseases, including cardiovascular and peripheral vascular toxicity, as well as Corrected QT (QTc) Interval prolongation (Lyon et al., 2022; Moslehi & Deininger, 2015; Sonnichsen et al., 2013). This led to a temporary withdrawal of ponatinib, in part due to severe cardiovascular side effects, followed by its reintroduction in 2014 with a restricted indication for T315I‐mutated leukemia (Goodrich, 2014; Cortes et al., 2012), indicating that ponatinib is a powerful yet potentially risky drug, and increasing its benefit‐to‐risk ratio is urgently needed.

In a study evaluating the cardiotoxic effects of imatinib and ponatinib on zebrafish embryos and rat H9c2 cardiomyoblasts, the viability of H9c2 cells was reduced, and zebrafish embryos exhibited dose‐dependent cardiac malformations. Both drugs were found to induce cardiac injury, with ponatinib demonstrating a stronger pharmacological effect (Zakaria et al., 2024). Ponatinib can activate the integrated stress response (ISR) in cardiac cells, leading to impaired cardiac function in mice, making it one of the most cardiotoxic TKIs (Yan et al., 2024). These direct damages on cardiac cells, leading to their reduced viability, suggest that there may be irreversible consequences of ponatinib‐induced toxicity. The mechanisms underlying ponatinib‐induced cardiac toxicity remain incompletely characterized. Therefore, investigating ponatinib‐induced cardiotoxicity holds significant importance for the prevention of significant adverse events.

Mechanistic studies have shown that ponatinib can downregulate the phosphorylation of AKT and ERK in zebrafish and rat cardiomyocytes, thereby interfering with signal transduction pathways and further affecting cell survival (Singh et al., 2019). Ponatinib can induce damage in human induced pluripotent stem cell‐derived cardiomyocytes (hiPSC‐CMs) and inhibit ATP synthase activity. Impaired ATP activated the general control nonderepressible 2 (GCN2)‐mediated integrated stress response (ISR), which contributed significantly to cardiac dysfunction (Yan et al., 2024). In studies investigating the in vivo antitumor efficacy of ponatinib, it was shown to suppress Programmed Death‐Ligand 1 (PD‐L1)‐related pathways by inhibiting the phosphorylation of MAPK, ERK, and JNK (Barnwal et al., 2023). Whether these pathways also play a role in patients' hearts remains unclear.

Current research on the mechanisms of ponatinib is based on models such as zebrafish, rat cardiomyocytes, hiPSC‐CMs, H9c2 cells, murine tumor cells and rat mesenteric artery endothelial cells (MAECs). These cells differ from adult human cardiomyocytes in terms of both species and maturity. Adult human primary cardiomyocytes (hPCMs) retain all natural physiological and pharmacological properties, and serve as a potentially ideal cellular model for cardiac physiology research, drug discovery, and safety evaluation. For example, hPCMs were used for the prediction of drug‐induced arrhythmogenic risk and mitochondrial injury, and exhibited superior performance, i.e., better clinical resemblance, compared to hiPSC‐CMs (Nguyen et al., 2017; Yusheng et al., 2018; Zhou et al., 2022). Therefore, investigating the molecular biological mechanisms of cardiotoxicity in hPCMs holds profound significance for cardiovascular research, drug discovery, and disease exploration.

In our previous work, we established a functionally competent and high‐fidelity hPCM model and developed high‐throughput mitochondrial toxicity detection methods, demonstrating that hPCMs exhibited excellent pharmacological response characteristics (Zhou et al., 2022). In this study, we compared the cellular responses of hPCMs and hiPSC‐CMs to ponatinib and found that, while both show increased apoptosis, the underlying cellular pathways are different. Specifically, whereas AKT activity was suppressed in both models, ERK activity was not impaired in hPCMs. AKT inhibitor MK‐2206 reduced the number of viable hPCMs, while the PI3Kα (phosphoinositide 3‐kinase alpha) activator UCL‐TRO‐1938 or insulin showed protective effects. These findings highlight the PI3Kα‐AKT pathway as a major pathway mediating the effect of ponatinib in adult human cardiomyocytes.

## 2 MATERIALS AND METHODS

### 2.1 Cell culture and reagents

In this study, all hPCMs were isolated from left atrial appendages of patients undergoing coronary bypass surgery. This study was approved by the Ethics Committee of Fuwai Hospital, Chinese Academy of Medical Sciences, Peking Union Medical College (No. 2021‐1533), and conformed to the Declaration of Helsinki. Written informed consent was obtained from all patients. A total of 36 patients were included, comprising 6 females and 30 males, with an average age of 62 years. The isolation and culture of hPCMs was performed as previously described (Zhou et al., 2022). Briefly, the left atrial appendages from patients were quickly minced into small pieces and subjected to enzymatic digestion using protease XXIV (Sigma‐Aldrich, P8038) and collagenase type II (Worthington, LS004196). After digestion, hPCMs were collected by centrifugation (100 × *g*, 3 min, 4°C), and calcium chloride was gradually reintroduced to a final concentration of 1.8 mmol L^−1^. Cells were seeded at densities of 13 × 10^4^, 2.5 × 10^4^, and 5500 cells per well in 12‐well (Corning, CLS3513), 24‐well (Corning, CLS3473), and 384‐well (Corning, CLS3765) plates for Western blotting, cell staining, and concentration–response/viability assays, respectively. Cells were cultured in Minimum Essential Medium (MEM) (Gibco, 42360099) supplemented with 1% bovine serum albumin (MP Biomedicals, 0219989680), 100 U mL^−1^ penicillin–streptomycin (Gibco, 15140163), 100 μg mL ^−1^ Primocin® (InvivoGen, ant‐pm‐2) and 10 μmol L^−1^(−)‐blebbistatin (Selleck, S7099) at 37°C with 5% CO_2_.

HiPSC‐CMs were derived from clones 1–1 (male), 1–2 (male), 5–1 (male), and 6–4 (male), with an average age of 57 years, used in our previous study (Zhou et al., 2022). The differentiation of undifferentiated hiPSCs into hiPSC‐CMs was performed as previously described (Zhou et al., 2022). hiPSC‐CMs were derived from clones 1–1, 1–2, 5–1, and 6–4, with all clones used within 45–70 days of differentiation to assess PON‐induced cardiotoxicity. For downstream experiments, hiPSC‐CMs were plated at a density of 80 × 10^4^ cells per well in 12‐well plates. Cells were cultured in RPMI 1640 medium (Gibco, 11875093) supplemented with serum‐free B27™ Supplement (1×, Gibco, 17504044) at 37°C in a humidified atmosphere of 5% CO_2_/95% air.

### 2.2 Concentration‐response curve

Concentration ‐response experiments were performed in 384‐well plates with 4 replicate wells for each concentration. Drugs were diluted to the intended concentration in culture medium containing 0.1% DMSO. After 48 h of drug treatment, cell viability was assessed using the CellTiter‐Glo® Luminescent Cell Viability Assay Kit (Promega, G7570) following the manufacturer's instructions. Luminescence was measured using a microplate reader (Tecan, Infinite 200 pro). Raw plate data from each titration point were normalized to the control. The normalized data were fitted to a four‐parameter Hill equation using the “log(inhibitor) vs. response – Variable slope (four parameters)” model in GraphPad Prism (version 8.2.1) to determine the half‐maximal inhibitory concentration (IC_50_). The equation used was Y = Bottom + (Top – Bottom)/(1 + 10^((LogIC_50_ – X) × HillSlope)), where X is the logarithm of concentration, Y is the inhibitory response, and the parameters Top, Bottom, LogIC_50_, and HillSlope correspond to the maximum response, minimum response, midpoint log‐concentration, and slope factor, respectively. These four parameters were derived by the software through nonlinear regression analysis.

### 2.3 Cell viability

After 48 h of drug treatment, cell medium was aspirated, and cell viability was measured using the LIVE/DEAD® Viability/Cytotoxicity Kit (ThermoFisher, L3224), following the manufacturer's instructions. Images were captured using an inverted fluorescence microscope (ZEISS, Axio Observer D1) at 20× magnification (microscope objective LD Plan‐Neofluar 20×/0.4 Korr Ph 2 M27), and cell counts were performed. Cell viability was quantified by manually counting live (green) and dead (red) cells in three random, independent images per group, using the ImageJ software (v1.8.0). Cell viability was calculated as follows: Cell viability = number of live cells/total cell number × 100%. Round cells displaying bright green fluorescence were considered apoptotic and thus were not counted as viable cells. Cell size measurements were also performed using ZEN 2 (Carl Zeiss, 2.0.0.0 blue edition).

Alternatively, cell viability was also measured using the CellTiter‐Glo® Luminescent Cell Viability Assay Kit (Promega, G7570). After drug treatment, cells were plated in 384‐well plates with 4–8 replicate wells per group. Following 48 h of cell culture, 25 μL of CellTiter Glo reagent was added to each well. The plate was shaken for 2 min, and luminescence was measured using a microplate reader (Tecan, Infinite 200 pro) after 10 min. Experiments were repeated with samples from 5 (Figure 3) or 4 (Figure 4) independent patient donors, respectively.

### 2.4 Western blot

Proteins were extracted using radioimmunoprecipitation assay (RIPA) buffer containing protease (BIOSS, C05‐01002) and phosphatase inhibitor (Thermo, A32957) cocktails (Zhou et al., 2022). The concentration of extracted proteins was determined using the Thermo BCA Protein Assay Kit (Thermo, 23225). A total of 20–25 μg of protein was loaded onto NuPAGE Bis‐Tris (Thermo, NP0322BOX) precast gels, and separated using a Mini Gel Tank system (Invitrogen). Proteins were transferred onto polyvinylidene difluoride (PVDF) membranes. After blocking at room temperature for 1.5 h, the membranes were incubated with the following primary antibodies (all diluted in TBST unless otherwise specified) overnight at 4°C: anti‐p‐AKT^S473^ (CST, 4060S, 1:1000), anti‐p‐AKT^T308^ (CST, 13038T, 1:1000), anti‐AKT (CST, 4691S, 1:1000), anti‐p‐p38 MAPK (CST, 4511T, 1:1000), anti‐p38 MAPK (CST, 8690T, 1:1000), anti‐p‐ERK (CST, 4370S, 1:1000), anti‐ERK (CST, 4695S, 1:1000), anti‐cleaved‐caspase‐3 (CST, 9661S, 1:1000), and GAPDH (Proteintech, 60004‐1‐1g, 1:10000). After three washes with TBST, the membranes were incubated with HRP‐conjugated secondary antibodies (CST, 7076S and CST, 7074S). Following another three washes with TBST, the membranes were developed using a 1:1 mixture of chemiluminescent substrates A and B (Thermo, 34096), and protein signals were detected using a western blot imaging system (Amersham ImageQuant 800 imager, Japan). Protein expression was quantified using ImageJ software (v1.8.0). Western blots were biologically repeated 3–5 times using either cells from different patients (hPCMs), or hiPSC‐CMs derived from the same or different hiPSC clones.

### 2.5 Statistical analysis

All experiments were performed with at least three independent replicates. For hPCMs, experiments were biologically repeated with samples from different patients; while for hiPSC‐CMs, biological repeats were performed using either the same clones or separate clones. Data are presented as mean ± standard error of the mean (SEM). Statistical analyses were performed using GraphPad Prism (version 8.2.1). All results are derived from at least three independent samples. Comparisons between two groups were performed using Student's *t*‐test. For comparison of paired data between three or more groups, we used repeated measures one‐way ANOVA with Dunnett's multiple comparison test. Exact *p‐values* are shown in the figures when *p* > 0.0001; ns, not significant.

## 3 RESULTS

### 3.1 Ponatinib induces cell death in human induced and adult cardiomyocytes

**FIGURE 1:**
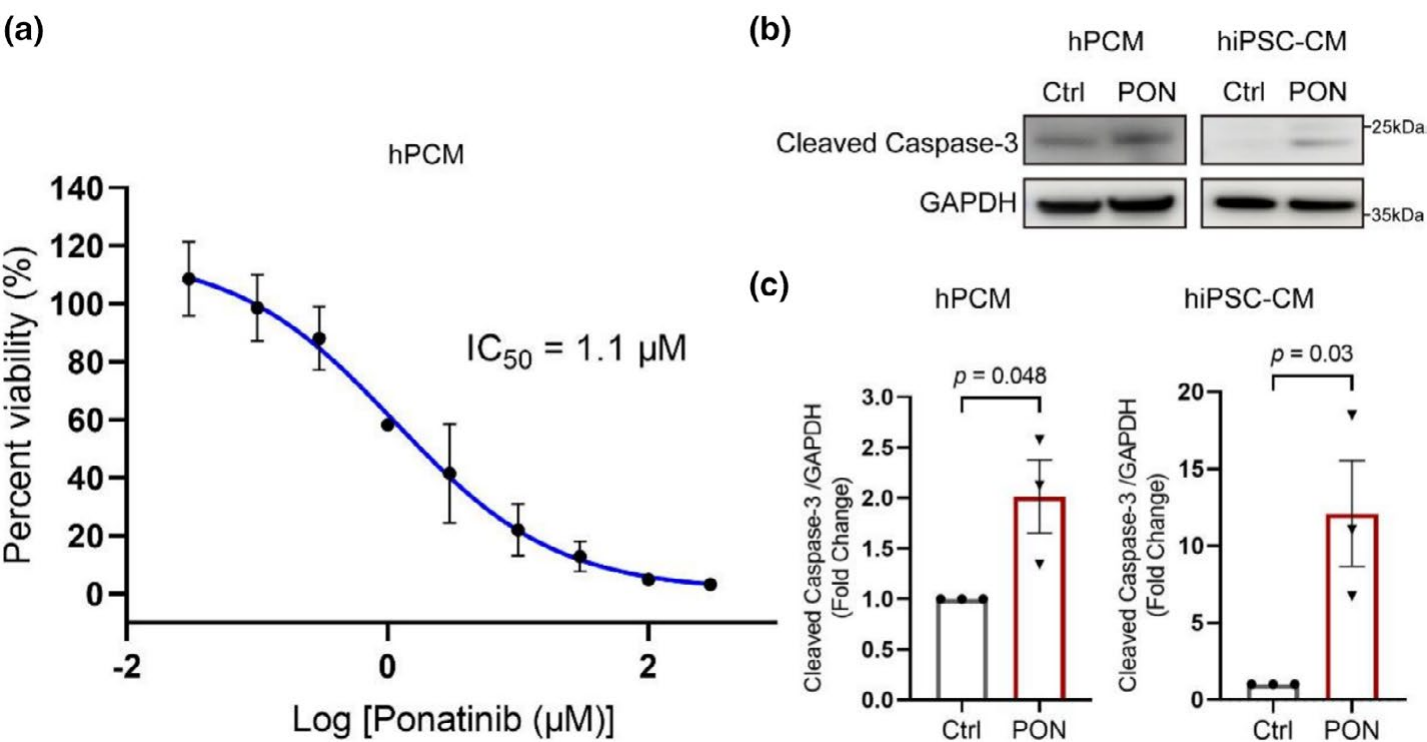
Ponatinib (PON) induces death in hPCMs and hiPSC‐CMs. (a) Concentration–response curve of hPCMs treated with ponatinib for 48 h. (b) Representative Western blots of cleaved caspase‐3 expression in hPCMs (left) and hiPSC‐CMs (right) treated with 1 μM PON for 2 h, with GAPDH as the loading control. (c) Quantification of (b), *n* = 3 independent experiments. Data are mean ± SEM; significance was calculated using unpaired Student's *t*‐test.

To assess the cellular response of hPCMs to ponatinib, we performed a concentration‐response experiment using 10 different doses of the drug. The calculated IC_50_ was 1.1 μM, suggesting cellular toxicity (Figure 1a). Treatment of hPCMs and hiPSC‐CMs with 1 μM ponatinib for 2 h significantly increased the level of cleaved caspase 3 in both cardiomyocyte models (Figure 1b–c). These results show that ponatinib confers cellular toxicity in human cardiomyocytes.

### 3.2 Ponatinib inhibits AKT signaling in cardiomyocytes

**FIGURE 2:**
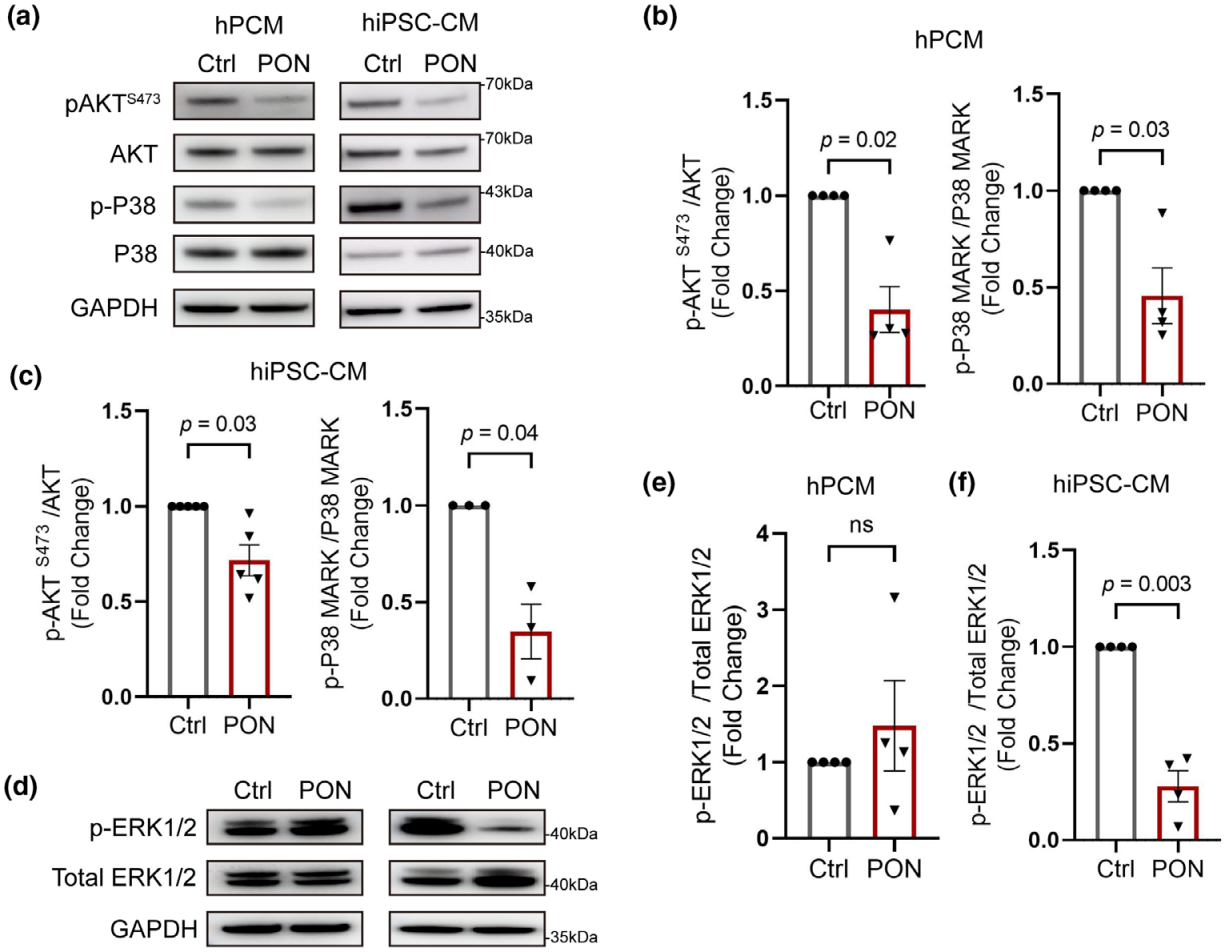
PON suppresses the PI3Kα‐AKT pathway. (a) Representative Western blots of p‐AKT^S473^ and p‐P38 MAPK in hPCMs (left) and hiPSC‐CMs (right) treated with 1 μM PON for 2 h, with GAPDH as the loading control. (b‐c) Quantification of p‐AKT^S473^ and p‐P38 in hPCMs (*n* = 4, b) and hiPSC‐CMs (*n* = 5 for p‐AKT^S473^ and *n* = 3 for p‐P38, c). (d) Representative Western blots of p‐ERK1/2 in hPCMs (left) and hiPSC‐CMs (right). (e, f) Quantification of p‐ERK1/2 in hPCMs (*n* = 4, e) and hiPSC‐CMs (*n* = 4, f). Data are mean ± SEM, significance was calculated using paired Student's *t*‐test.

To determine the effect of ponatinib on survival‐ and stress‐related signaling, we examined alterations in the AKT, ERK, and p38 signaling pathways in both hPCMs and hiPSC‐CMs. AKT Ser473 phosphorylation was significantly reduced in response to ponatinib treatment in both cell models, suggesting impaired survival signaling (Figure 2a–c). However, phosphorylation at T308 remained unchanged (Figure S1). Unexpectedly, p38 phosphorylation, a stress‐response signal, was also suppressed upon ponatinib treatment (Figure 2a–c). By contrast, ERK signaling was markedly suppressed in hiPSC‐CMs, but not in hPCMs, suggesting differential response mechanisms in these two cardiomyocyte models (Figure 2d–f).

### 3.3 AKT inhibitor MK‐2206 phenocopies ponatinib‐induced death of adult cardiomyocytes

**FIGURE 3:**
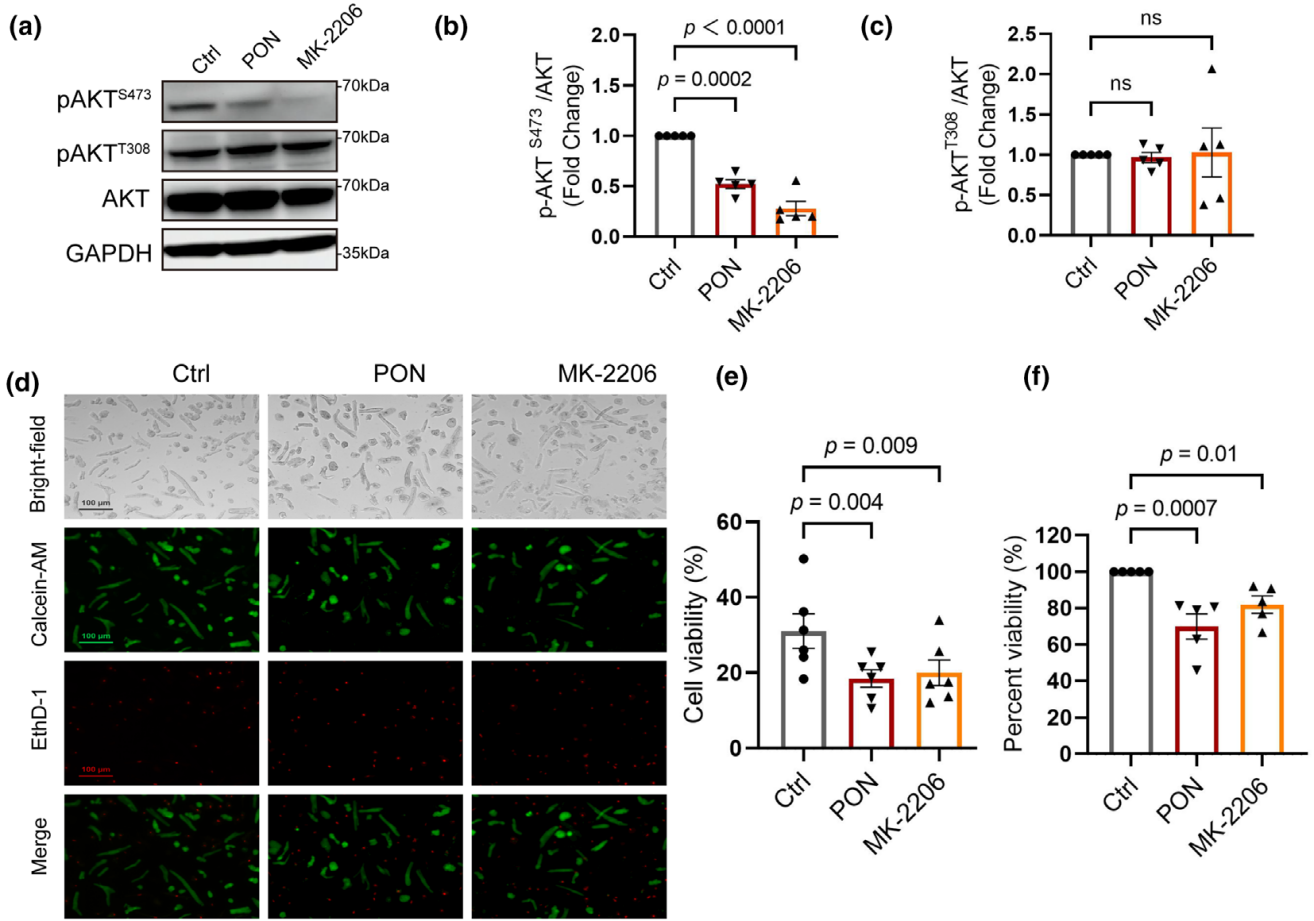
MK‐2206 induces cell death in hPCMs. (a) Western blotting of AKT phosphorylation (S473 and T308) in hPCMs treated with 5 μM PON or 10 μM MK‐2206 for 48 h, with GAPDH as the loading control. (b, c) Quantification of p‐AKT^S473^ (b) and p‐AKT^T308^ (c) in (a), *n* = 5. (d) Live/dead staining of hPCMs. Green (Calcein‐AM) indicates live cells, red (EthD‐1) indicates dead cells, and the DMSO group served as the control. Scale bar = 100 μm. (e) Quantification of (d), *n* = 6 in each group. (f) Cell viability assay of hPCMs (*n* = 5). Data are presented as mean ± SEM, data in b, c, e, and f were analyzed by repeated measures one‐way ANOVA, followed by Dunnett's multiple comparison test.

We thus reasoned that ERK and p38 signaling are unlikely to play a role in ponatinib‐induced hPCM cell death. Therefore, we asked whether AKT inhibition was central to hPCM survival. To this end, we used a known inhibitor of AKT, MK‐2206. Like ponatinib, MK‐2206 strongly inhibited AKT Ser473 phosphorylation in hPCMs, but not T308 phosphorylation (Figure 3a–c). We then used calcein and ethidium homodimer to evaluate cell viability of hPCMs. As expected, both ponatinib and MK‐2206 significantly reduced cell viability (Figure 3d,e). Alternatively, we used an ATP‐based method to assess cell survival. Consistent with staining results, both ponatinib and MK‐2206 significantly reduced cell survival (Figure 3f). Collectively, these results suggest that hPCMs' survival is dependent on AKT signaling, which was suppressed by ponatinib.

### 3.4 AKT activation protects from ponatinib‐induced cardiomyocyte death

**FIGURE 4:**
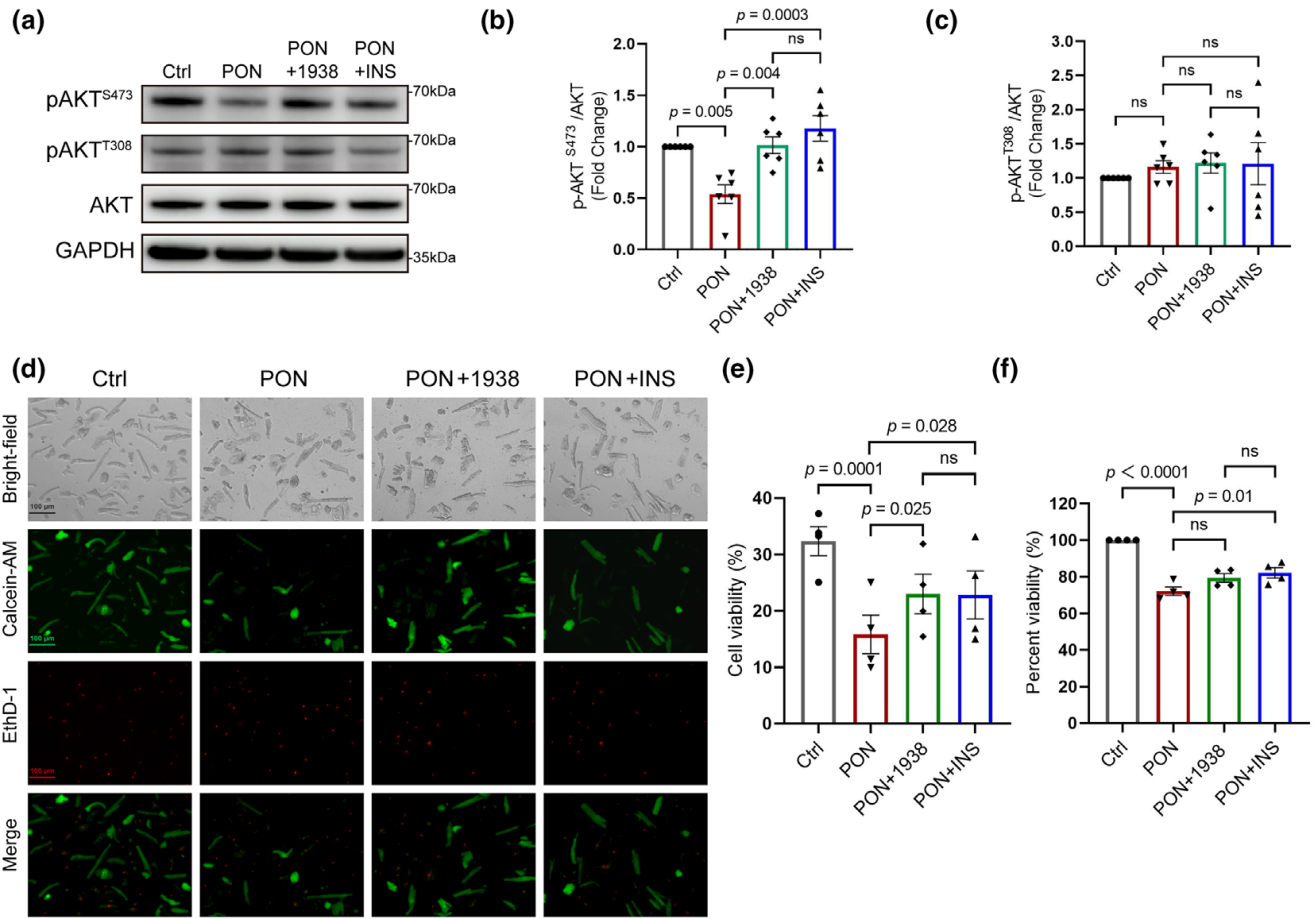
Activation of PI3Kα‐AKT pathway attenuates PON‐induced cardiomyocyte death. (a) Western blotting of AKT phosphorylation (S473 and T308) in hPCMs pretreated with 0.1 μM UCL‐TRO‐1938 or 1 μM insulin (INS) for 1 h, followed by exposure to 5 μM PON for 48 h, with GAPDH as the loading control. (b, c) Quantification of p‐AKT^S473^ (b) and p‐AKT^T308^ (c) in (a), *n* = 6. D, Live/dead staining of hPCMs. Green (Calcein‐AM) indicates live cells, red (EthD‐1) indicates dead cells. Scale bar = 100 μm. (e) Quantification of (d), *n* = 4. (f), Cell viability assay of hPCMs (*n* = 4). Data are presented as mean ± SEM. Data in b, c, e, and f were analyzed by repeated measures one‐way ANOVA, followed by Dunnett's multiple comparison test.

Based on the above findings, we hypothesized that AKT activation would protect from cardiomyocyte death. Therefore, we treated cells with ponatinib in conjunction with insulin, a known activator of the PI3Kα‐AKT pathway, or UCL‐TRO‐1938, a recently discovered allosteric activator of PI3Kα (Gong et al., 2023). Pretreatment of hPCMs with these activators for 1 h prevented the inhibition of AKT signaling upon ponatinib treatment (Figure 4a–c). Insulin modestly, but significantly, improved cell viability as assessed by both calcein staining and ATP‐based measurement (Figure 4d–f). UCL‐TRO‐1938 also significantly improved cell viability as measured by CellTiter Glo, and showed an increasing trend using calcein staining (Figure 4d–f). Together, these findings show that UCL‐TRO‐1938 and insulin confer partial protection against ponatinib‐induced cardiomyocyte death.

## 4 DISCUSSION

This study is the first to utilize hPCMs to investigate cardiomyocyte toxicity of ponatinib, potentially providing new insights into toxicity prevention and treatment. Importantly, ERK1/2 signaling was reported to exert an important role in mediating ponatinib toxicity in hiPSC‐CMs, zebrafish, and isolated neonatal rat cardiomyocytes (Singh et al., 2019; Talbert et al., 2015), while our study indicated that it may not be a central regulator in adult human cardiomyocytes. It has been reported that ponatinib induces paradoxical activation of the MAPK pathway via BRAF‐CRAF dimerization, resulting in ERK1/2 activation in other model systems, which may explain this unexpected observation (Wilhelm et al., 2023). By contrast, AKT signaling was shown to be a critical mediator of such toxicity, whose restoration mitigated cell death. This also illustrates the differential response of two well‐known pathways, which are often observed to act in parallel in cardiac contexts (Klawitter et al., 2018; Sbroggiò et al., 2011; Solan et al., 2019; Wang et al., 2022).

Ponatinib is a multi‐target kinase inhibitor, primarily targeting the Bcr‐Abl tyrosine kinase. It was also reported to inhibit more than 60 kinases (Singh et al., 2020), including fibroblast growth factor receptors (FGFR 1–4), receptor interacting serine/threonine kinase 2 (RIPK2), FMS‐like tyrosine kinase 3 (FLT3), platelet‐derived growth factor receptor α (PDGFRα), KIT proto‐oncogene, receptor tyrosine kinase (c‐KIT), and Rearranged during Transfection (RET) (Gao et al., 1878). By doing so, it potently inhibits AKT, which is critical for killing cancer cells. However, our study illustrated the importance of AKT for the survival of cardiomyocytes, which are the major functional units of the heart. While activating AKT did prevent cardiomyocyte death, it may also dampen the effect of ponatinib on cancer cells. In the future, this dilemma may be solved by using organ‐specific targeting and delivery techniques, such as ligand conjugation (Fu & Xiang, 2020; Han et al., 2021; Zhao et al., 2020; Zinger et al., 2020).

Currently, the toxic effects of ponatinib on other cardiac cell types, for example on cardiac microvascular endothelial cells, or cardiac fibroblasts, are incompletely understood. In vivo, ponatinib was shown to increase blood pressure, impair endothelium‐dependent relaxation (EDR), and cause injury to endothelial cells in SD rats (Tang et al., 2025). It also activated endothelial cells, platelets, and leukocytes, driving plaque inflammation and leading to death from myocardial infarction and stroke in mice (Stepanian et al., 2025). Another form of vascular toxicity produced by ponatinib was VWF‐mediated platelet adhesion, which triggered microvascular angiopathy and subsequent left ventricular wall motion abnormalities (Latifi et al., 2019). In vitro, ponatinib induces toxicity in hiPSC‐CFs and hiPSC‐ECs (Sharma et al., 2017).

In summary, the toxic mechanisms of ponatinib on cardiomyocytes are highly complex, involving various signaling pathways. Our results highlight the AKT pathway as a major target mediating the toxic effects of ponatinib, resulting in cell death of adult human primary cardiomyocytes, which can be ameliorated by activation of AKT signaling. These findings provide insight into ponatinib toxicity that may be directly clinically relevant and offer guidance for developing novel cardioprotective strategies.

## 5 CONCLUSION

The results demonstrate that ponatinib induces apoptosis in hPCMs via inhibition of AKT signaling, while small molecule‐ and insulin‐induced AKT activation offers a feasible approach for protecting hPCMs.

## AUTHOR CONTRIBUTIONS

**Bingying Zhou:** Conceptualization; funding acquisition; methodology; project administration; resources; supervision. **Linna Guo:** Data curation; formal analysis; investigation; methodology; validation; visualization. **Rongjia Rao:** Investigation. **Hong Liu:** Investigation.

## FUNDING INFORMATION

This work was supported by the National High Level Hospital Clinical Research Funding (grant 2022‐GSP‐TS‐9 to B.Z.), and the National Natural Science Foundation of China (grant 82070287 to B.Z.).

## CONFLICT OF INTEREST STATEMENT

The authors have declared that no conflict of interest exists.

## ETHICS STATEMENT

Written informed consent was obtained from all patients. This study was approved by the Ethics Committee of Fuwai Hospital, Chinese Academy of Medical Sciences, Peking Union Medical College (2021‐1533), and conformed to the Declaration of Helsinki.

## INFORMED CONSENT

Written informed consent was obtained from all patients.

## Supporting information


Figure S1.

## Data Availability

Authors will provide all raw data associated with this manuscript upon request. Data is available in Excel, and all full unaltered immunoblots are available upon request.
