# Supplementary material for: Ponatinib confers adult human cardiomyocyte toxicity via inhibition of AKT signaling
Source: Physiol Rep. 2026 Apr 17;14(8):e70877. doi: 10.14814/phy2.70877 (PMC13088335; doi:10.14814/phy2.70877)
Supplement: Supplementary file 1 — Figure S1. [file PHY2-14-e70877-s001.docx]

Supplemental Figure S1


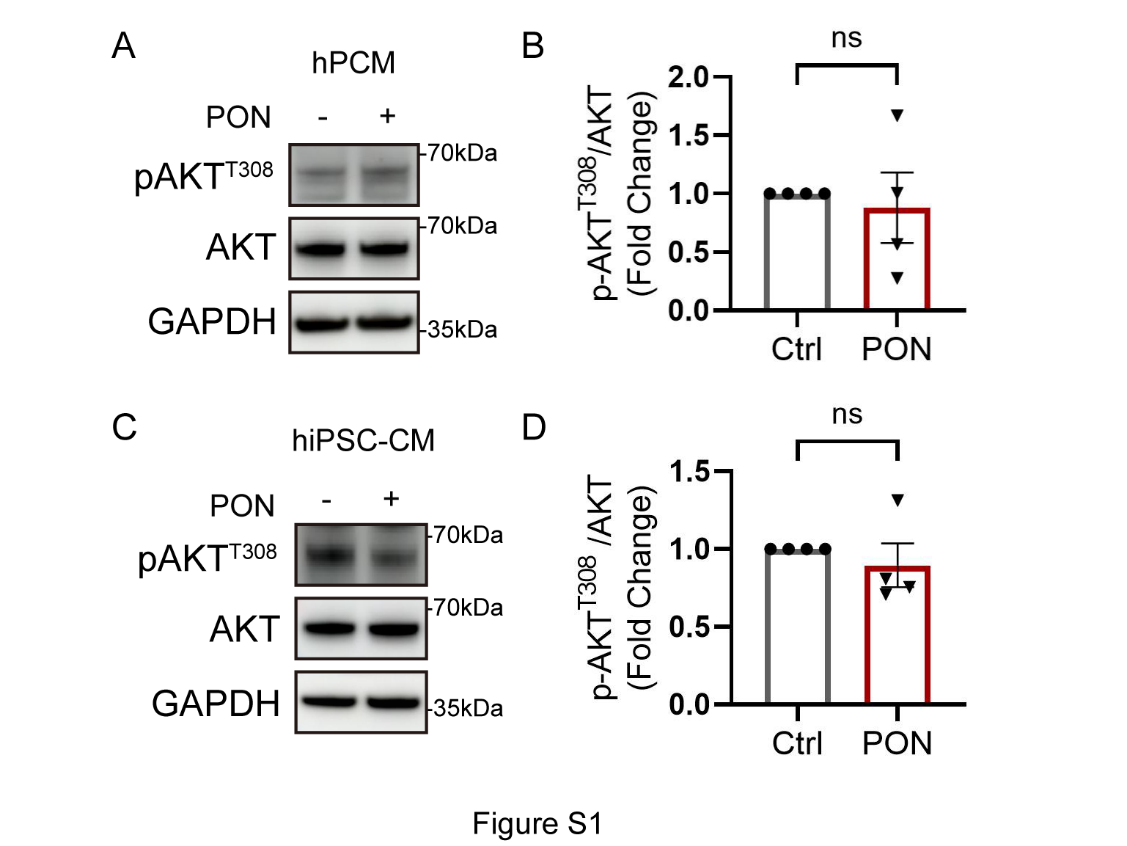


**Supplemental Figure S1**. A, Representative Western blots of p-AKT(T308) in hPCMs treated with 1 μM PON for 2 h, with GAPDH as the internal reference. B, Quantification of (A), n = 4 independent experiments. C, Representative Western blots of p-AKT(T308) in hiPSC-CMs treated with 1 μM PON for 2, with GAPDH as the internal reference. D, Quantification of (C), n = 4 independent experiments. Data are presented as mean ± SEM, and statistical analysis was performed using paired Student's *t*-test.
